# Reliability and validity of a new physical activity questionnaire for India

**DOI:** 10.1186/s12966-015-0196-2

**Published:** 2015-03-18

**Authors:** Ranjit Mohan Anjana, Vasudevan Sudha, Nagarajan Lakshmipriya, Sivasankaran Subhashini, Rajendra Pradeepa, Loganathan Geetha, Mookambika Ramya Bai, Rajagopal Gayathri, Mohan Deepa, Ranjit Unnikrishnan, Valsalakumari Sreekumaran Nair Binu, Anura V Kurpad, Viswanathan Mohan

**Affiliations:** Madras Diabetes Research Foundation & Dr. Mohan’s Diabetes Specialities Centre, WHO Collaborating Centre for Non-communicable Diseases Prevention and Control, IDF Centre of Education, 6B, Conran Smith Road, Gopalapuram, 600086 Chennai India; Department of Statistics, Manipal University, Manipal, 576104 Karnataka India; Department of Nutrition, St. John’s Research Institute, St. John’s National Academy of Health Sciences, Bangalore, 560 034 Karnataka India

**Keywords:** Physical activity, Exercise, GPAQ, IPAQ, India, Questionnaire

## Abstract

**Background:**

Measurement of physical activity in epidemiological studies requires tools which are reliable, valid and culturally relevant. We attempted to develop a physical activity questionnaire (PAQ) that would measure physical activity in various domains over a year and which would be valid for use in adults of different age groups with varying levels of activity in urban and rural settings in low and middle income countries like India. The present paper aims to assess the reliability and validity of this new PAQ- termed the Madras Diabetes Research Foundation- Physical Activity Questionnaire (MPAQ).

**Methods:**

The MPAQ was administered by trained interviewers to 543 individuals of either gender aged 20 years and above from urban and rural areas in 10 states of India from May to August 2011, followed by a repeat administration within a month for assessing reliability. Relative validity was performed against the Global Physical Activity Questionnaire (GPAQ). Construct validity was tested by plotting time spent in sitting and moderate and vigorous physical activity (MVPA) against body-mass index (BMI) and waist circumference. Criterion validity was assessed using the triaxial accelerometer, in a separate subset of 103 individuals. Bland and Altman plots were used to assess the agreement between MPAQ and accelerometer.

**Results:**

The interclass correlation coefficients (ICC) for total energy expenditure and physical activity levels were 0.82 and 0.73 respectively, between baseline and 1st month. The ICC between GPAQ and the MPAQ was 0.40 overall. The construct validity of the MPAQ showed linear association between sitting and MVPA, and BMI and waist circumference independent of age and gender. The Spearman’s correlation coefficients for sedentary activity, MVPA and overall PA for MPAQ against the accelerometer were 0.48 (95%CI-0.32-0.62), 0.44 (0.27-0.59) and 0.46 (0.29-0.60) respectively. Bland and Altman plots showed good agreement between MPAQ and accelerometer for sedentary behavior and fair agreement for MVPA.

**Conclusion:**

The MPAQ is an acceptable, reproducible and valid instrument, which captures data from multiple activity domains over the period of a year from adults of both genders and varying ages in various walks of life residing in urban and rural India.

**Electronic supplementary material:**

The online version of this article (doi:10.1186/s12966-015-0196-2) contains supplementary material, which is available to authorized users.

## Introduction

Physical inactivity has been recognized as a major modifiable risk factor for non-communicable diseases (NCDs) since the 1950s [1]. Recent reports have equated the impact of physical activity (PA) to that of smoking with respect to the worldwide burden of NCDs [2]. Physical activity is a challenging variable to measure, on account of the inherent complexity and diversity of human behavior. Traditionally, tools for measuring physical activity have been divided into subjective and objective methods. While the use of objective methods generally provides more accurate estimates of physical activity, these methods are cumbersome and impractical for use outside the setting of specialized research units. Subjective (self-reported) methods involving the use of physical activity questionnaires (PAQs) have therefore become the preferred method of assessing physical activity in epidemiological studies.

A number of PAQs have been described in the literature, most of which have been designed for use, and validated in, developed countries. Several factors mitigate against the use of these questionnaires in low and middle income countries like India. A major drawback of these PAQs in the Indian context, is the importance given to leisure time physical activity (LTPA). While LTPA contributes significantly to total physical activity in Western populations, studies from India show that less than 10% of the population performs any LTPA at all [3]. Also, the use of many of these PAQs demands a certain level of literacy in the respondents, which may not be the case in developing countries like India.

In recent years, international questionnaires such as the Global Physical Activity Questionnaire (GPAQ) [4] and International Physical Activity Questionnaire (IPAQ) [5] have been validated in several populations, including those of developing nations. Many of these questionnaires, though valid and reliable, do not permit collection of information on region-specific and culturally relevant activities across different domains. These questionnaires assess physical activity over the week prior to administration and may not be suited for use in individuals with varied educational levels as seen in India, as they require the respondent to self-rate their own level of activity intensity, which has been shown to be difficult in the Indian setting.

The Indian Migration Study (IMS) questionnaire [6] was developed as an alternative to the international questionnaires for use in India. While the IMS questionnaire is reliable, valid and culturally relevant, it only collects information pertaining to the month immediately preceding its administration. Also, the IMS questionnaire does not address the aspect of seasonality of occupations and variations in physical activity in individuals holding multiple jobs at the same time.

Therefore, we attempted to develop a PAQ for use in India, that would measure habitual, culturally relevant activities in various domains (occupational, transport, recreational, activities of daily living and weekend activities) over a year and which would be valid for use in adults of different age groups with varying levels of activity in urban as well as rural settings. The present paper aims to assess the reliability and validity of this new PAQ- termed the Madras Diabetes Research Foundation- Physical Activity Questionnaire (MPAQ).

## Methodology

The MPAQ was developed (Additional file 1) after reviewing various published validated physical activity questionnaires both in India and abroad. In addition, 24 hour physical activity recalls encompassing a weekday and weekend were collected from 50 volunteers across all ages and occupations. From these 24 hr recalls, the various activities reported across all domains were listed in the MPAQ and similar activities were grouped together and further truncated based on the average energy cost, as the Physical Activity Ratio (PAR) provided by the WHO/FAO 2001 [7]. The MPAQ was designed to capture frequency and duration of habitual obligatory and discretional activities by means of a mix of open and closed-ended questions arranged in four domains viz. work-related activity (work domain), activities of daily living [general activity domain which includes sleep (daytime napping and sleep at night), personal care and domestic chores], transport-related activities (transport domain) and recreational activities (recreational domain). In all domains, options are provided to capture both seasonal and non-seasonal activities. The questionnaire captures details of up to two jobs and elicits information on time spent sitting, standing, walking and climbing stairs in each of these jobs, providing insight into the nature of the job and intensity of work activity.

In the recreational domain, the questionnaire elicits information on sedentary behavior (including TV viewing, chatting with friends, listening to music etc.) as well as light, moderate and vigorous activities on a daily, weekly or monthly basis. In addition, there is provision for recording the extra activities or extra hours of sedentary behavior that happen during the weekend. The questionnaire enables calculation of physical activity for an “average” day by summing up activities in various domains for a 24-hour period. Similarly, weekly and monthly calculations can also be done and information aggregated to compute activity for a year.

Total energy expenditure can be estimated through factorial calculations recommended by a joint FAO/WHO/UNU expert consultation [7]. The factorial calculations are based on the time spent on various activities in the multiple domains and the energy cost of these activities. Energy cost is reported as a multiple of Basal Metabolic Rate (BMR) and called Physical Activity Ratio (PAR). Total time spent on habitual activities is multiplied by PAR to derive the total energy expenditure (TEE) of 24 hours. The physical activity level (PAL) can then be calculated as TEE/BMR for 24 hours. Based on the PAL values [7], individuals can be divided into three categories: Sedentary (1.40 – 1.69), moderately active (1.70-1.99) and vigorously active (2.00-2.40) [7].

Written informed consent was obtained from each participant before start of the reliability and validity studies. Institutional Ethics Committee approval was obtained from the Ethics Committee at MDRF.

### Reliability study

**Figure 1:**
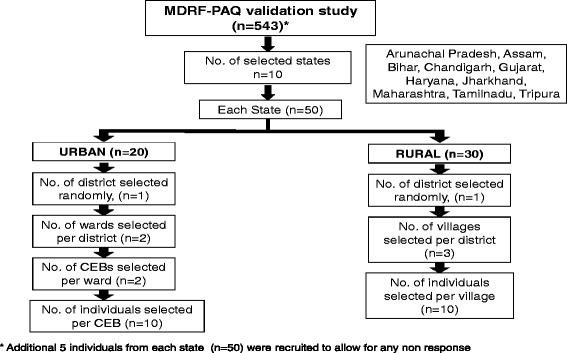
Selection of study subjects.

The MPAQ was administered by trained interviewers to individuals of either gender aged 20 years and above from 10 states in India namely Tamilnadu, Gujarat, Maharashtra, Jharkhand, Haryana, Bihar, Chandigarh, Assam, Tripura and Arunachal Pradesh. The states were so chosen as to be representative of the country in terms of geography, socioeconomic status, variability of occupations and climatic conditions. From one district in each state, two census enumeration blocks (CEBs) in urban areas and three villages in rural areas were randomly selected (Figure 1). In each CEB or village, 10 households were randomly selected and in each household, one individual was selected to participate in the study. Thus, 50 individuals were selected from each state, and 500 for the entire study. In addition, five individuals from each state were recruited to allow for non-response over time. Hence a total of 550 individuals were initially invited for the study, of whom 543 individuals participated and had all required information at baseline. The participants were sampled so as to obtain individuals across all age categories and both genders with varying literacy levels and engaged in a wide range of occupations so as to test the ability of the MPAQ to measure physical activity of individuals from all walks of life.

Demographic details and information on smoking and alcohol use were obtained from all participants as well as height, weight, waist and blood pressure (BP) measurements, assessed using standardized techniques. Weight (in kilograms-kg) was measured with the subjects wearing light clothing after having removed shoes and heavy jewelry. Height was measured to the nearest centimeter (cm) using a stadiometer with the subjects standing erect without shoes. Body mass index (BMI) was calculated as the weight (kg) divided by the height (in meters squared). Waist circumference was measured using a non-stretchable tape, as the mean of two measurements of the smallest horizontal girth between the costal margins and the iliac crests at minimal respiration. BP was recorded in the sitting position in the right arm using an electronic instrument (Model: HEM- 7101, Omron Corporation, Tokyo, Japan). Two readings were taken 5 minutes apart and the mean of 2 readings was taken as the blood pressure.

The baseline administration of MPAQ was performed from May to August 2011 in all the states. This was followed by a repeat administration within a month for assessing reliability. The interval of one month was chosen based on a previously published study from India [6], and was deemed most appropriate to eliminate recall of previous responses by the participants as well as any possibility of physical activity patterns having significantly altered in the interim.

### Validity studies

For assessing relative validity, the MPAQ and the GPAQ were administered in a randomized order by trained interviewers to all selected participants across the 10 states one day apart so as to avoid questionnaire fatigue. The GPAQ was chosen because it is a widely used global PAQ which has been validated in India [8]. The test questionnaire took on an average, 10 minutes (±5) to administer. Subject acceptability and co-operation were good with both questionnaires but the subject understanding was better with the test questionnaire.

Construct validity indicates the consistency or the relationship between the activity instrument (MPAQ) and the physiological variable such as BMI. This was tested by plotting time spent in sitting and moderate and vigorous physical activity (MVPA) (measured as minutes/day) against BMI and waist circumference measured at baseline.

For assessing criterion validity, 107 individuals of either gender aged 20 years and above were recruited from Chennai city in Tamilnadu. The sample was so chosen as to get individuals across a wide age range, both genders and all categories of activity: At the start of the study, information on demographic parameters, height, weight and occupation were obtained as described above. Criterion validity was assessed using the Actigraph (Actilife 5) GT3X+ Triaxial Accelerometer (Actigraph, Pensacola, Florida, USA). Participants were asked to wear the accelerometer for 7 days during waking hours; however, the device was allowed to be removed while bathing or swimming. The device was worn on the hip of the dominant side (right in most cases). The device was worn either above or beneath clothing and not necessarily in contact with skin; however, a snug fit against the body was ensured to avoid erroneous readings.

Accelerometers were initialized to monitor and record data in 60- second “epochs” as “activity counts” and sample frequency at 100 Hz. The start date and time and stop date and time were used for the start and stop of data collection. While initializing, each device was given a unique number denoting the individual participant with their age, gender, height, weight, date of birth and race.

The GT3X+ device collects data from all three axes of movement regardless of the configuration, with Axis 1 collecting the vertical axis acceleration activity data, Axis 2 the horizontal axis data and Axis 3 the perpendicular axis data. The duration (minutes per day) spent in different intensity activities- light (1.5-3 METS, 100 ≤ 1951 counts), moderate (3–6 METS, 1952–5724 counts) and vigorous (>6 METS, ≥ 5725 counts) were determined according to published data [9,10].

The MPAQ was administered anytime during the period the individual was wearing the accelerometer. Data from the MPAQ was computed for a typical week, and then converted to minutes/week, so as to make comparisons with the accelerometer data more realistic.

### Inter-rater reliability

MPAQ being an interviewer administered questionnaire, inter rater reliability was measured to assess the agreement between the interviewers. One interviewer administered the questionnaire to the participant while the rest of the interviewers passively observed and rated participant’s response independently. This procedure was completed for a total of 20 participants by all 20 interviewers who collected the questionnaires across the 10 states. A kappa value of 0.83 indicated good agreement among the interviewers.

### Statistical analyses

Statistical analyses were performed using a SAS (Statistical Analysis System) statistical package (version 9.0; SAS Institute, Inc., Cary, NC). The results are expressed as mean ± standard deviation or proportions.

Reliability of the MPAQ was examined by calculating the intra class correlation coefficient (ICC) of the activities reported and presented by urban/rural status and gender. ICC values of <0.40 were considered as poor agreement, 0.40-0.59 as fair, 0.60-0.74 as good and 0.75-1.0 as excellent agreement [11]. Relative validity between the GPAQ and MPAQ was also assessed using ICC.

Construct validity was used to assess the degree to which a measure (in this case, the MPAQ) compares with an underlying theoretical construct (a latent variable in this context such as BMI and waist circumference). Linear regression models were used to assess the association of deciles/tertiles of activity with BMI and waist circumference after adjusting for age and gender.

For assessing criterion validity, the MPAQ was compared against the triaxial accelerometer as a criterion. Spearman’s correlation coefficients and 95% CI were used for comparisons. Total duration (minutes/week) of time spent in sedentary and MVPA as estimated from the MPAQ were compared against those recorded by the accelerometer using established cut-points [10]. As the accelerometer measured data for a week, the data obtained from the MPAQ was also computed for a week so as to make it comparable. Accelerometer data were initially downloaded and processed using customized software viz. Actilife Data Analysis Software [Version 5.0], prepared by Actigraph R&D and Software department (Florida, USA). For the purposes of this study, correlation coefficient values: < 0.20, 0.21-0.40, 0.41-0.60, 0.61-0.80 and 0.81-1.0 were considered as weak, fair, moderate, strong and very strong correlation respectively [6].

Bland and Altman plots were used to assess the agreement between data obtained using the MPAQ and accelerometer (within the 95% limits). In addition this plot also indicates the random and systematic errors of the data. The mean difference (bias) between accelerometer and the MPAQ, of sedentary activity/ week were plotted (y- axis) against the mean of estimated sedentary minutes/week obtained from the accelerometer and MPAQ [12]. A similar plot was constructed for MVPA minutes as well. A p-value <0.05 was considered significant for all statistical measures.

## Results

Of the 543 selected participants in the reliability study, 288 were male (53%). Tables 1 and 2 show the baseline characteristics of the study population state-wise, in urban and rural areas respectively. In urban areas, the overall mean age was 44 ± 14 years, BMI was 23.7 ± 4.2 kg/m^2^, waist 83.5 ± 12.1 cm, SBP 134 ± 20 mm Hg and DBP 81 ± 12 mmHg. Overall, 82% of the population was literate, 19% smoked and 21% consumed alcohol. In rural areas, the mean age was 42 ± 13 years, BMI 21.9 ± 4.2 kg/m^2^, waist 78.3 ± 11 cm, systolic BP 129 ± 19 mm Hg and diastolic BP 79 ± 11 mm Hg. 74% were literate, 16% smoked and 20% consumed alcohol.

**Table 1:** **Baseline characteristics of the urban population studied (State Wise)**

**Variables**	**Chandigarh**	**Haryana**	**Bihar**	**Arunachal Pradesh**	**Tripura**	**Assam**	**Jharkhand**	**Gujarat**	**Maharashtra**	**Tamil Nadu**	**Overall**
N	20	20	21	16	17	19	21	20	20	35	209
Age (years)	42.8 ± 3.9	44.3 ± 3.8	40.8 ± 2.9	45.1 ± 3.2	45.4 ± 2.8	44.2 ± 4.0	46.2 ± 3.9	37.8 ± 5.6	46.4 ± 4.8	48.3 ± 4.7	44.4 ± 14.2
BMI (kg/m^2^)	24.5 ± 11.4	25.9 ± 12.4	22.4 ± 13.6	23.1 ± 14.1	22.8 ± 12.7	22.1 ± 17.7	24.3 ± 14.8	25.5 ± 12.7	21.7 ± 14.8	24.2 ± 15.7	23.7 ± 4.2
Waist (cms)	87.0 ± 11.0	86.6 ± 11.2	81.7 ± 9.9	80.4 ± 7.9	84.8 ± 8.9	80.4 ± 9.9	89.4 ± 14.3	82.6 ± 15.6	78.0 ± 11.7	83.5 ± 13.7	83.5 ± 12.1
SBP (mmHg)	139 ± 11	134 ± 9	131 ± 13	134 ± 13	130 ± 12	136 ± 7	134 ± 11	132 ± 11	123 ± 13	138 ± 16	134 ± 20
DBP (mmHg)	80 ± 20	81 ± 15	81 ± 22	84 ± 23	83 ± 15	79 ± 22	78 ± 20	80 ± 15	76 ± 16	84 ± 25	81 ± 12
Literate n(%)	15 (75.0)	18 (90.0)	12 (57.1)	10 (62.5)	14 (82.4)	17 (89.5)	17 (81.0)	17 (85.0)	18 (90.0)	33 (94.3)	171 (81.8)
Smoking n(%)	3 (15.0)	7 (35.0)	7 (33.3)	3 (18.8)	5 (29.4)	1 (5.3)	4 (19.0)	1 (5.0)	2 (10.0)	6 (17.1)	39 (18.7)
Alcohol n(%)	6 (30.0)	3 (15.0)	4 (19.0)	7 (43.8)	1 (5.9)	2 (10.5)	6 (28.6)	0 (0.0)	8 (40.0)	6 (17.1)	43 (20.6)

SBP- Systolic blood pressure; DBP- Diastolic blood pressure; BMI-Body Mass Index.

**Table 2:** **Baseline characteristics of the rural population studied (State Wise)**

**Variables**	**Chandigarh**	**Haryana**	**Bihar**	**Arunachal Pradesh**	**Tripura**	**Assam**	**Jharkhand**	**Gujarat**	**Maharashtra**	**Tamil Nadu**	**Overall**
N	30	30	31	37	26	24	32	30	30	64	334
Age (years)	43.7 ± 5.1	44.0 ± 5.8	39.9 ± 3.0	38.5 ± 3.7	40.7 ± 3.7	37.9 ± 2.9	45.8 ± 3.6	39.5 ± 3.4	45.1 ± 3.7	41.7 ± 4.1	41.7 ± 13.4
BMI (kg/m^2^)	24.2 ± 11.6	23.5 ± 13.7	20.8 ± 15.8	22.7 ± 11.2	21.7 ± 14.5	19.7 ± 11.8	20.1 ± 11.9	22.1 ± 10.9	20.1 ± 16.3	22.6 ± 14.1	21.9 ± 4.2
Waist (cms)	84.0 ± 13.5	83.9 ± 14.7	75.6 ± 10.5	80.6 ± 7.0	79.4 ± 10.0	72.6 ± 6.8	74.8 ± 10.0	79.4 ± 9.3	72.1 ± 8.9	79.1 ± 11.8	78.3 ± 11.2
SBP (mmHg)	128 ± 12	130 ± 13	130 ± 11	130 ± 12	127 ± 9	132 ± 12	127 ± 10	127 ± 11	125 ± 11	132 ± 11.0	129 ± 18.8
DBP (mmHg)	79 ± 15	80 ± 23	77 ± 23	84 ± 21	76 ± 15	80 ± 23	77 ± 15	81 ± 13	79 ± 17	79 ± 20	79 ± 11.4
Literate n (%)	25 (83.3)	23 (76.7)	13 (41.9)	26 (70.3)	20 (76.9)	18 (75.0)	19 (59.4)	22 (73.3)	22 (73.3)	60 (93.8)	248 (74.3)
Smoking n (%)	5 (16.7)	5 (16.7)	1 (3.2)	10 (27.0)	9 (34.6)	2 (8.3)	5 (15.6)	5 (16.7)	6 (20.0)	6 (9.4)	54 (16.2)
Alcohol n (%)	10 (33.3)	5 (16.7)	2 (6.5)	16 (43.2)	7 (26.9)	3 (12.5)	11 (34.4)	1 (3.3)	8 (26.7)	2 (3.1)	65 (19.5)

SBP- Systolic blood pressure; DBP- Diastolic blood pressure; BMI-Body Mass Index.

Table 3 shows the results of the reliability study. The maximum time spent was in the work domain both in urban and rural areas as well as among males and females. Out of the 543 participants at baseline, 520 were available in the 1st month (95.7%). Overall the ICC for TEE and PAL between the baseline and the 1st month were 0.82 and 0.73 respectively, demonstrating good reliability of the MPAQ. The table also presents the ICC domain wise. The lowest ICC was for recreational activity (0.58), and the highest for sitting (0.81). The reliability was good in both males and females and in both urban and rural areas.

**Table 3:** **Reliability study: ICC of MPAQ at baseline and 1 month**

**Domains/Activities**	**Overall**			**Male**			**Female**			**Urban**			**Rural**		
	**Time spent (mins/d)**		**ICC**	**Time spent, (mins/d)**		**ICC**	**Time spent, (mins/d)**		**ICC**	**Time spent, (mins/d)**		**ICC**	**Time spent** **(mins/d)**		**ICC**
	**Base line, N = 543**	**1 month, N = 520**		**Base line, N = 288**	**1 month, N = 277**		**Base line, N = 255**	**1 month, N = 243**		**Base line, N = 209**	**1 month, N = 199**		**Base line, N = 334**	**1 month, N = 321**	
Work	507.1 ± 212.2	490.5 ± 222.7	0.72	540.4 ± 191.8	524.2 ± 210.6	0.74	428.4 ± 237	408.8 ± 231.1	0.63	546.5 ± 258.1	535.2 ± 268.8	0.79	482.2 ± 173.3	463 ± 184.4	0.61
Transport	135.3 ± 59.2	130.6 ± 66.4	0.67	159.5 ± 61.0	149.2 ± 71.3	0.62	108.0 ± 43.0	109.2 ± 52.7	0.61	123.5 ± 53.4	119.9 ± 56.1	0.63	142.7 ± 61.5	137.1 ± 71.3	0.72
Sleeping	370.2 ± 55.7	358.06 ± 56.8	0.63	368.9 ± 55.3	362.5 ± 56.1	0.60	371.7 ± 56.3	353 ± 57.2	0.67	380.2 ± 47.5	371.9 ± 53.4	0.64	364 ± 59.5	349.5 ± 57.2	0.61
General activity*	199.9 ± 116.3	173.8 ± 97.9	0.75	132.3 ± 66.2	118.0 ± 66.4	0.59	276.2 ± 113.4	237.5 ± 88.7	0.61	211.1 ± 119.2	178.5 ± 106.5	0.75	192.9 ± 114	171 ± 92.2	0.74
Recreation#	254.7 ± 111.1	241.4 ± 115.8	0.58	238.0 ± 106.7	237.3 ± 111.4	0.61	273.6 ± 113.1	246.2 ± 120.7	0.57	256.7 ± 115.3	240.1 ± 115.6	0.64	253.5 ± 108.6	241.7 ± 116.1	0.54
Walking as an exercises	48.4 ± 38.4	52.4 ± 45.4	0.68	53.7 ± 43.1	58.8 ± 47.9	0.72	42.4 ± 32.7	45.1 ± 42.6	0.54	30.9 ± 24.8	37.38 ± 29.2	0.71	59.3 ± 42.1	61.6 ± 51.6	0.65
Sitting	176.9 ± 152.7	190.8 ± 163.8	0.81	213.5 ± 160.1	229.3 ± 166.4	0.83	134.8 ± 132.0	146.7 ± 149.4	0.74	224.0 ± 187.2	231 ± 192.6	0.85	148.01 ± 118.3	165.9 ± 137.7	0.73
TV viewing	127.4 ± 55.6	120.7 ± 57.9	0.67	119 ± 53.4	118.7 ± 55.7	0.71	136.8 ± 56.7	123.1 ± 60.3	0.63	128.4 ± 57.6	120.5 ± 57.8	0.78	126.7 ± 54.3	120.9 ± 58.0	0.60
Total Energy Expenditure (TEE)	36.9 ± 11.4	35.1 ± 12.1	0.82	39.6 ± 11.7	38.7 ± 12.6	0.86	33.8 ± 10.4	31.1 ± 10.0	0.74	36.6 ± 11.7	34.9 ± 12.7	0.82	37.04 ± 11.3	35.20 ± 11.7	0.81
Physical Activity Level (PAL)^$$^	1.56 ± 0.47	1.47 ± 0.49	0.73	1.67 ± 0.48	1.61 ± 0.53	0.75	1.44 ± 0.43	1.30 ± 0.40	0.65	1.56 ± 0.47	1.47 ± 0.50	0.78	1.57 ± 0.47	1.47 ± 0.51	0.70

*Include obligatory personal and domestic chores ^$$^PAL- Physical Activity Level. ^#^Recreation includes sitting, TV viewing and walking as an exercises.

The relative validity between the already validated GPAQ and MPAQ was evaluated. Of the 543 participants administered the MPAQ, data from GPAQ was available in 440. The ICC between GPAQ and the MPAQ was 0.40 overall, indicating fair correlations between the questionnaires. The ICC was found to be highest for sitting (0.57) followed by MVPA recreational (0.50), work vigorous (0.46) and transport (0.44). (Data not shown).

**Figure 2:**
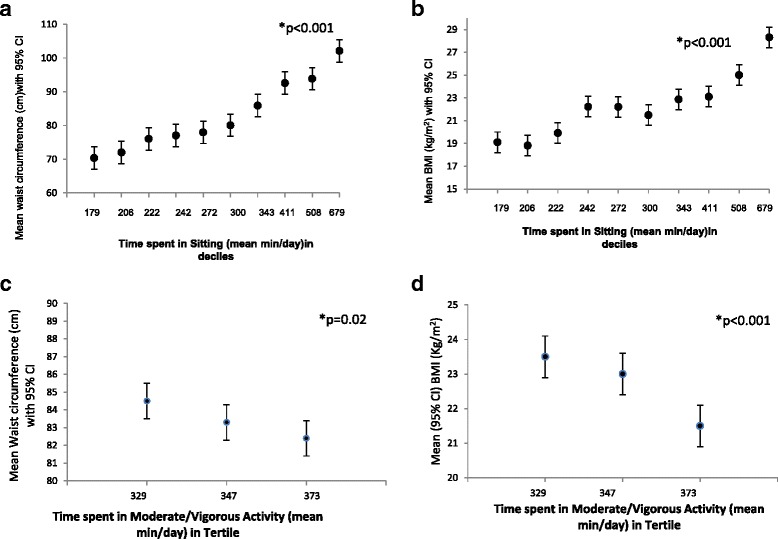
Construct validity of the MPAQ (a-d). **a** Association of waist circumference with deciles of time spent in sitting(means and 95% confidence intervals), *p value adjusted for age and sex. **b** Association of BMI with deciles of time spent in sitting(means and 95% confidence intervals), *p value adjusted for age and sex. **c** Association of waist circumference with tertiles of time spent in moderate/vigorous activity (means and 95% confidence intervals), *p value adjusted for age and sex. **d** Association of BMI with tertiles of time spent in moderate/vigorous activity (means and 95% confidence intervals) *p value adjusted for age and sex.

Figure 2 shows the construct validity of the MPAQ. Figure 2a to d show the association of sitting and MVPA measured by the MPAQ with BMI and waist circumference independent of age and gender. Individuals in the lowest decile of sitting had the lowest waist circumference (mean difference between highest decile and lowest decile - 32.1 cm, 95%CI −35.0 to - 29.1; p value <0.001) (Figure 2a) and BMI (mean difference between highest decile and lowest decile −9.3 kg/m^2^, 95%CI −10.42 to −8.15; p value <0.001) (Figure 2b) compared to those in the highest decile whereas those in the highest tertile of MVPA had the lowest waist circumference (highest tertile versus lowest tertile mean difference in WC 3.47 cm, 95%CI 0.50-6.44; p value =0.02), a 2.5% decrease in WC (Figure 2c) and lowest BMI (highest tertile versus lowest tertile mean difference in BMI 2.3 kg/m^2^, 95%CI 1.41-3.20; p value <0.001), a 9.5% decrease in BMI (Figure 2d).

For criterion validity, after excluding individuals with missing data and incorrect usage of accelerometer, data from 103 individuals was available for analysis. Data was considered acceptable if it covered activity over five full days (10 hours/day) including a weekend day. Of the 102 participants included in the criterion validity study, 54 (52.4%) were male. The mean age of the participants was 32 ± 8.7 years and the mean BMI, 25.2 ± 4.5 kg/m^2^.

Table 4 shows the correlation between physical activity assessed by the MPAQ and measured by the accelerometer. The Spearman’s correlation coefficients for sedentary activity, MVPA and overall physical activity for MPAQ against the accelerometer were 0.48 (95% CI-0.32-0.62), 0.44 (0.27-0.59) and 0.46 (0.29-0.60) respectively, showing modest correlation of the MPAQ with the reference method (accelerometer).

**Table 4:** **Spearman’s correlation between accelerometer and MPAQ (n = 103)**

**Variables**	**Accelerometer (mins/week)**	**MPAQ (mins/week)**	**Spearman’s correlation**	
	**Mean ± SD**	**Mean ± SD**	**r**	**95% CI**
Sedentary activity	5009 ± 785	4965 ± 869	0.484	0.32-0.62
Moderate and Vigorous physical activity	252 ± 208	350 ± 206	0.443	0.27-0.59
Total physical activity	7120 ± 76.8	7110 ± 197	0.458	0.29-0.60

**Figure 3:**
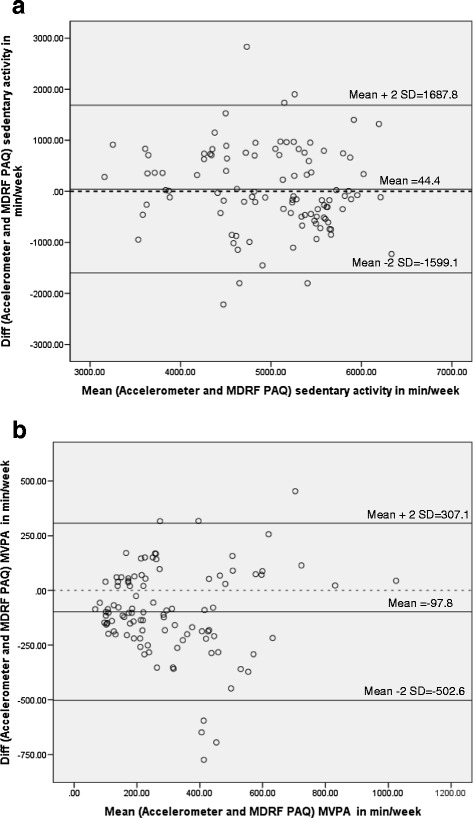
Bland-Altman plots showing the agreement between MPAQ and accelerometer. **a:** Agreement for sedentary activity (n=103). **b:** Agreement for moderate-to-vigorous activity (n=103).

Figure 3a and b present the Bland and Altman plots representing the agreement between sedentary activity and MVPA obtained from the MPAQ (measured as minutes/week) against the accelerometer (also measured as minutes/week). Figure 3a shows that the agreement between the MPAQ and the accelerometer for sedentary behavior was good [mean bias = 44.4 minutes/week, ±2SD −1599 to 1688] min/week. A similar plot was seen for MVPA [mean bias = 97.8, ±2SD −502.6 to 307.1] min/week (Figure 3b).

## Discussion

The MPAQ was developed with the objective of producing an instrument which can be used widely in epidemiological studies in India. One of the main advantages of the MPAQ is that it assesses physical activity patterns over the period of a year, enabling it to take into consideration, the variability in activity patterns that occurs over this time period. Other physical activity questionnaires capture activities over a typical week or month, which may be unduly influenced by the time at which the questionnaire is administered and may not be truly representative of the individual’s overall levels of physical activity over longer periods of time. This is especially so in rural areas where agricultural activities are inherently seasonal in nature. Even in urban areas, individuals could have more than one job at a time, either full-time or part-time. Others might pursue one form of occupation during the week and another during the weekend. The MPAQ attempts to address the issue of variability in physical activity patterns by asking specifically about seasonality and nature of occupations performed and listing in detail the number of months in a year, weeks in a month, days in a week and hours in a day each specific job is performed (for up to two jobs). It also specifically looks at time spent in walking, sitting, standing etc. for each of the jobs performed.

The MPAQ also had good reliability at 1 month from baseline with an ICC of 0.82 which is comparable to other widely accepted PAQs, such as the EPIC- Norfolk questionnaire, GPAQ, IPAQ and IMS questionnaires, all of which have reported reliability values varying from 0.67 to 0.88 [6,8,13-16]. Questionnaires assessing past year activity have also shown similar estimates of repeatability [17-20]. The reliability was best for sitting and lowest for the recreational domain, which could perhaps be a reflection of the magnitude of the activities performed.

In the relative validity study, the MPAQ showed moderate correlation (ICC 0.40) with the GPAQ in all the domains. The absence of a stronger correlation is not surprising since the two questionnaires are inherently different in many respects. For instance, the GPAQ does not capture details on many activities culturally relevant to India that are included in the MPAQ. Even in domains that are common (i.e., transport domain), walking and cycling are captured together as one question in GPAQ in contrast to the MPAQ, where these are captured separately, thus making comparisons difficult.

The construct validity study used categories of physical activity based upon reported time in different activity intensities to predict association with anthropometric indices such as waist circumference and BMI. Individuals who performed the most physical activity had the lowest waist circumference and BMI, thereby providing a more robust assessment of validity of the MPAQ. However, as in any cross-sectional study, these results must considered in light of the probability of reverse causation, that is, individuals becoming less active on account of the physical and psychological constraints imposed by overweight and obesity.

For measuring criterion validity, we used the triaxial accelerometer as the reference method. While tools such as indirect calorimetry, doubly labelled water method and heart rate monitoring have been considered as the gold standard for measures of energy expenditure, logistics precluded the use of these techniques in the present study. Accelerometers are a less expensive and more convenient alternative for the objective measurement of physical activity and have been widely used for this purpose [8,13]. Our results show that the relationship was good for comparisons of total activity, sedentary behaviour and MVPA measures obtained using the MPAQ and the triaxial accelerometer. This is in line with other questionnaires such as the EPIC-Norfolk questionnaire, GPAQ, IPAQ, IMS questionnaire etc. which have been evaluated for criterion validity against different reference methods and have correlation coefficients ranging from 0.28 to 0.48 [6,9,14].

This newly developed physical activity questionnaire provides a practical method to assess physical activity for risk stratification of chronic diseases in large observational studies and epidemiological surveillance. This questionnaire captures various dimensions of physical activity such as the type, intensity and duration and estimates the time spent in these activities, and hence could rank individuals based on the physical activity level. Thus this questionnaire could be recommended for studies that assess the health consequences and correlates of physical activity or lack thereof.

The MPAQ also has some limitations. Firstly, as for any questionnaire, it is subject to recall bias, which could lead to overestimation of activity levels in some domains and underestimation in others. Also, recent events are likely to be recalled more accurately than those in the distant past, introducing another element of bias. The questionnaire only calculates physical activity for an “average” day, week or month; physical activity assessments for a specific day, week or month by month cannot be obtained. In addition, in the Bland Altman- the mean values were a small fraction of the magnitude of the measurement, indicating that the MPAQ is a better tool for large epidemiological studies rather than for individual assessments. However, it was also seen that the MPAQ over-reports MVPA and under-reports sedentary behavior, to a minimal extent. This observation is perhaps due to the small number of subjects engaged in MVPA and the relatively low volume of MVPA performed by these subjects. Moreover, physical activity questionnaires have been shown to be prone to both random and systematic errors, with respondents tending to over-report physical activity and under-report sedentary behavior that are influenced by cultural and social desirability factors [15]. In additions the questionnaire also requires adequate training and understanding in order to obtain good quality data.

To conclude, our results show that the MPAQ is a relevant, acceptable, reproducible and valid instrument, which, in a single administration, captures data from multiple activity domains over the period of a year from adults of both genders and varying ages in various walks of life residing in urban and rural areas of different regions of the country. The MPAQ is also the first to have been validated in the north-eastern region of India, which differs significantly from the rest of the country in terrain, climatic conditions and lifestyles, all of which can alter physical activity patterns. In addition, the MPAQ is also easy to administer and well-understood by the study subjects. The MPAQ can therefore be considered an important addition to the physical activity researcher’s armamentarium in that it helps to overcome many of the challenges of measuring physical activity in low and middle income countries like India, making assessment of this important risk factor easier and perhaps, more accurate.

## Additional file

Additional file 1:
**MDRF Physical activity questionnaire (MPAQ).**
