# Supplementary material for: Reliability and validity of a new physical activity questionnaire for India
Source: Int J Behav Nutr Phys Act. 2015 Mar 18;12:40. doi: 10.1186/s12966-015-0196-2 (PMC4498505; doi:10.1186/s12966-015-0196-2)
Supplement: Additional file 1: — MDRF Physical activity questionnaire (MPAQ). [file 12966_2015_196_MOESM1_ESM.pdf]

MDRF PHYSICAL ACTIVITY QUESTIONNAIRE (MPAQ)

Name : ______________________________ **Volunteer code** ☐☐☐☐☐☐

Age: ☐☐☐ **yrs** **Sex: M** ☐ **F** ☐ **Interview** ☐☐ ☐☐ ☐☐☐☐
Date Month Year

Height: ☐☐☐.☐ **cms** **Weight:** ☐☐☐.☐ Kgs **Body fat:** ☐☐.☐ %

BP: ☐☐☐ **/** ☐☐☐

In this section, you will be asked about the time spent doing different types of physical activity. Please answer these questions even if you do not consider yourself to be a physically active person			
**SECTION I – PHYSICAL ACTIVITY AT WORK:** *This section applies only to those who are employed and students. Housewives, retired people and those who are unemployed (for 1 year) can skip this section and go to (Q. no 13ai).*			
1	Does your work involve Seasonal Activity		Yes 1<br>No 2 ☐
2a	Specify Primary or Full Time Occupation: ______________________________		
2b	Specify Seasonal* or Part-time Occupation**, if any ____________________(Write 99 if not applicable)		
	Indicate your duration of occupation **<u>per year</u>**		
3a	Full Time Occupation<br>Months ☐☐ /Year	3b	Seasonal* / Part-Time Occupation**<br>Months ☐☐ Year
	Indicate your duration of occupation **<u>per Week</u>**		
4a	Full Time Occupation<br>Days ☐ / Week **OR**<br>Days ☐☐ / Month	4b	Seasonal* /Part-Time Occupation**<br>Days ☐ / Week **OR**<br>Days ☐☐ / Month
	On an average, how many hours **<u>per day</u>** do you spend at work place?		
5a	Full Time Occupation<br>☐☐ ☐☐ / Day<br>Hours : Minutes	5b	Seasonal */ Part Time Occupation**<br>☐☐ ☐☐ / Day<br>Hours : Minutes

*Seasonal - Typically reflects changes in occupation due to monsoon or winter
**Part-time - The second job

	At the work place, how many hours per day do you spend on the following:				
**Full Time Occupation / Day**		**Duration (Hrs: mins)**	**Seasonal* / Part –Time Occupation** / Day**		***Duration (Hrs: mins)***
6a	Skilled occupational activities – (professional activities like crèche care taker, heavy vehicle driving, tailoring, laundering, house-keeping etc.,)	☐☐ ☐☐	6b	Skilled occupational activities - (professional activities like crèche care taker, heavy vehicle driving, tailoring, laundering, house-keeping etc.,)	☐☐ ☐☐
7a	An activity more strenuous than walking (like planting, manual agricultural work, carrying heavy load, professional sports person etc.,)	☐☐ ☐☐	7b	An activity more strenuous than walking (like planting, manual agricultural work, carrying heavy load, professional sports person etc.,)	☐☐ ☐☐
8a	Standing	☐☐ ☐☐	8b	Standing	☐☐ ☐☐
9a	Walking at varying paces without a load	☐☐ ☐☐	9b	Walking at varying paces without a load	☐☐ ☐☐
10a	Climbing stairs / Walking uphill	☐☐ ☐☐	10b	Climbing stairs / Walking uphill	☐☐ ☐☐
11a	Light vehicle driving during office hours	☐☐ ☐☐	11b	Light vehicle driving during office hours	☐☐ ☐☐
12a	Sitting (Office work like typing, reading, computer work)	☐☐ ☐☐	12b	Sitting (Office work like typing, reading, computer work)	☐☐ ☐☐

*Seasonal - Typically reflects changes in occupation due to monsoon or winter
**Part-time - The second job

	**SECTION II – PHYSICAL ACTIVITY –GENERAL WEEKDAY (Other than work related)**<br>**In this section you will be asked about physical activity in general (other than work related) like domestic chores, personal care etc.,**											
**Q.no**	**Weekday activity - General**							13<br><br>14	Do you have seasonal* changes in activity?<br>Yes-1 No -2 :<br>If yes, for how many months does this change apply?			
	**Activity**	**Duration / day (Hrs: mins)**	**Daily**	**Weekly**	**Monthly**	**Yearly**	**Never**		**Duration / day (Hrs: mins)**	**Daily**	**Weekly**	**Monthly**
15a<br><br>16a	Sleeping (Regular hours of sleep usually at night) and Nap(short break of sleep - day time)	Night<br><br>Day						15b<br><br>16b	Night<br><br>Day			
17a	Personal care - brushing, toilet , showering, dressing etc.,							17b				
18a	Eating (Include all meals, snacks & coffee/tea drinks) except that reported in the work section							18b				
19a	Cooking – (including pre-preparation of meals, snacks and beverages)							19b				
20a	Collecting water/ wood (by manual means like well, hand pumping)							20b				
21a	Climbing steps / walking uphill							21b				
22a	Non-mechanized domestic chores (like -sweeping, washing clothes and dishes by hand)							22b				
23a	Toddler care (age < 5 years includes feeding, bathing and playing etc.,)							23b				

*Seasonal - Typically reflects changes in occupation due to monsoon or winter
**Part-time - The second job

SECTION III – PHYSICAL ACTIVITY- Commutation (WEEK DAYS)
In this section you will be asked about the usual way you travel to and from places like self-driving, cycling, commuting to different places etc.,

Q.no	Weekday activity - Transport							24<br>25	Do you have seasonal* changes in activity? Yes-1 No -2 :<br>If yes, for how many months does this change apply?			
	**Activity**	**Duration / day (Hrs: mins)**	**Daily**	**Weekly**	**Monthly**	**Yearly**	**Never**		**Duration / day (Hrs: mins)**	**Daily**	**Weekly**	**Monthly**
26a	Self driving–work and other places (car/bike/scooter)							26b				
27a	Commuting by bus / auto /pillion rider (to work & other places)							27b				
28a	Travel by cycling (excludes cycling as an exercise)							28b				
29a	Walking to and fro places (excludes walking as an exercise)							29b				

SECTION IV – PHYSICAL ACTIVITY- LEISURE (WEEK DAYS)
This section excludes the work and transport activities that you have already mentioned. You will be asked about sports, fitness and recreational activities (leisure) like watching TV, chatting, reading etc

Q.no	Weekday activity - Leisure							30<br>31	Do you have seasonal* changes in activity? Yes-1 No -2 :<br>If yes, for how many months does this change apply?			
	**Activity - Light**	**Duration / day (Hrs: mins)**	**Daily**	**Weekly**	**Monthly**	**Yearly**	**Never**		**Duration / day (Hrs: mins)**	**Daily**	**Weekly**	**Monthly**
32a	Slow walking (Example- Shopping, going to a worship place)							32b				
33a	Playing a musical Instrument/ Singing (as a hobby)							33b				
34a	Others specify ________________							34b				
35a	Others specify ________________							35b				

*Seasonal - Typically reflects changes in occupation due to monsoon or winter
**Part-time - The second job

	Activity -Moderate	Duration / day (Hrs: mins)	Daily	Weekly	Monthly	Yearly	Never		Duration / day (Hrs: mins)	Daily	Weekly	Monthly
36a	Brisk walking as an exercise							36b				
37a	Cricket							37b				
38a	Jogging / slow running							38b				
39a	Dancing / aerobics/ yoga (asanas)							39b				
40a	Swimming							40b				
41a	Cycling including static cycling							41b				
42a	Others specify ____________							42b				
43a	Others specify ____________							43b				
	**Activity - Vigorous**	**Duration / day (Hrs: mins)**	**Daily**	**Weekly**	**Monthly**	**Yearly**	**Never**		**Duration / day (Hrs: mins)**	**Daily**	**Weekly**	**Monthly**
44a	Conditioning exercises (like muscle strengthening exercises, using a rowing machine, free weights etc.							44b				
45a	Running / sprinting							45b				
46a	Football, basket ball, tennis, volleyball etc.,							46b				
47a	Others specify ____________							47b				
48a	Others specify ____________							48b				

*Seasonal - Typically reflects changes in occupation due to monsoon or winter
**Part-time - The second job

	Activity -Sedentary	Duration / day (Hrs: mins)	Daily	Weekly	Monthly	Yearly	Never		Duration / day (Hrs: mins)	Daily	Weekly	Monthly
49a	Watching TV	☐☐ ☐☐						49b	☐☐ ☐☐			
50a	Bhajans /prayer	☐☐ ☐☐						50b	☐☐ ☐☐			
51a	Watching movies/shows/ concerts	☐☐ ☐☐						51b	☐☐ ☐☐			
52a	Yoga as relaxation	☐☐ ☐☐						52b	☐☐ ☐☐			
53a	Chatting, reading, sitting, listening to music etc.,	☐☐ ☐☐						53b	☐☐ ☐☐			
54a	Others specify ________________	☐☐ ☐☐						54b	☐☐ ☐☐			
55a	Others specify ________________	☐☐ ☐☐						55b	☐☐ ☐☐			

	**SECTION V – PHYSICAL ACTIVITIY- WEEKEND ACTIVITY: List all the activities that you do in weekend and are not mentioned in the earlier**		
56	Week end includes 1 day --------1<br>1 ½ days----2<br>2 days ------3 ☐ / Week<br>**or**<br>Days ☐ /month		
	**WEEKEND –General Activity**	Duration /day (Hrs: mins)	Monthly (Indicate the frequency)
57	Regular hours of sleep usually at night) and	Night sleep ☐☐ ☐☐	
58	Nap(short break of sleep - day time)	Day sleep ☐☐ ☐☐	
59	Cooking	☐☐ ☐☐	
60	Collecting water/wood/climbing steps/walking uphill	☐☐ ☐☐	
61	Non-mechanized domestic chores (example-sweeping, washing clothes and dishes by hand)	☐☐ ☐☐	
62	Toddler care (age <5 years includes feeding, bathing, playing etc.)	☐☐ ☐☐	
63	Personal care (brushing, toilet, showering, dressing etc.)	☐☐ ☐☐	

	WEEKEND -Transport	Duration /day (Hrs: mins)	Monthly (Indicate the frequency)
64	Self driving –work and other places (car/bike/scooter)		
65	Commuting by bus / auto /pillion rider (to work & other places)		
66	Travel by cycling (excludes cycling as an exercise)		
67	Walking to and fro places (excludes walking as an exercise)		
	**WEEKEND – Leisure Light Activity**	Duration /day (Hrs: mins)	Monthly (Indicate the frequency)
68	Slow walking (example- shopping, going to a worship place)		
69	Playing a musical instrument/ singing (as a hobby)		
	**WEEKEND – Leisure Moderate Activity**	Duration /day (Hrs: mins)	Monthly (Indicate the frequency)
70	Brisk walking as an exercise		
71	Cricket		
72	Dancing / aerobics / yoga (asanam)		
73	Swimming		
74	Jogging / running		
75	Cycling including static cycling		
	**WEEKEND – Leisure Vigorous Activity**	Duration /day (Hrs: mins)	Monthly (Indicate the frequency)
76	Conditioning exercises e.g. muscle strengthening exercises like using a rowing machine / free weights etc.		
77	Running / sprinting		
78	Football, basket ball, tennis, volleyball etc.,		

	WEEKEND – Leisure Sedentary Activity	Duration /day (Hrs: mins)	Monthly (Indicate the frequency)
79	Watching TV	☐☐ ☐☐	
80	Bhajans/Prayer at temple	☐☐ ☐☐	
81	Watching movies at theatre	☐☐ ☐☐	
82	Yoga as relaxation	☐☐ ☐☐	
83	Chatting, reading, sitting, listening to music etc.,	☐☐ ☐☐	
	**WEEKEND – Leisure Other Activity**	Duration /day (Hrs: mins)	Monthly (Indicate the frequency)
84	---	☐☐ ☐☐	
85	---	☐☐ ☐☐	
86	---	☐☐ ☐☐	
87	---	☐☐ ☐☐	
88	**COMMENTS:**		

Interviewer name and code -- ☐☐☐☐

Verified & Approved by (Field Supervisors):----------------------------- ☐☐☐☐

Date keyed on: -- ☐☐ ☐☐ ☐☐☐☐

*Seasonal - Typically reflects changes in occupation due to monsoon or winter
**Part-time - The second job
